# Screening for Caregiver Stress in an Urban Medical Home for Children with Medical Complexity: Results of a Pilot Study

**DOI:** 10.3390/children12040434

**Published:** 2025-03-29

**Authors:** Courtney L. Horton, Julie E. Heier, John R. Barber, Nicola Brodie

**Affiliations:** 1 Children’s National Hospital, Washington, DC 20010, USA; chorton@cnmc.org; 2 Division of General and Community Pediatrics, Children’s National Hospital, Washington, DC 20010, USA; jheier@cnmc.org; 3 Division of Psychiatry and Behavior Health, Children’s National Hospital, Washington, DC 20010, USA; 4 School of Medicine and Health Sciences, George Washington University, Washington, DC 20037, USA; 5 Division of Biostatistics and Study Methodology, Children’s National Hospital, Washington, DC 20010, USA

**Keywords:** children with medical complexity, caregiver mental health, caregiver stress, caregiver stress screening, social drivers of health, universal mental health screening, patient-centered medical home, childhood opportunity index

## Abstract

Background: Children with medical complexity (CMC), a subset of children with special healthcare needs, have chronic conditions affecting multiple organ systems, require medical technology, and account for a significant share of pediatric healthcare spending despite comprising only 1% of the population. Their families experience unique stressors, including financial strain and high rates of workforce attrition, suggesting medical inequity is an independent risk factor for health inequity. The role of universal caregiver stress screening using a validated tool within the outpatient primary care medical home for CMC youth has not been explored in the literature. Methods: Caregivers of all patients in the Complex Care Program (CCP) within a large academic pediatric primary care Medical Home-certified practice at the Children’s National Hospital were screened for caregiver stress during routine primary care appointments using the University of Washington Caregiver Stress Scale 8-Item Short Form V. 2.0 (UW-CSS). Elevated scores prompted referrals to the CCP psychosocial team, and composite scores were recorded in the electronic medical record. Demographics, medical diagnoses, and technology support status were extracted from the medical chart. The childhood opportunity index (COI) was calculated as a proxy for socioeconomic position. Results: Screening for caregiver stress in our medical home for CMC was feasible and yielded unexpected results. We found no difference in levels of stress among caregivers based on the COI. This finding highlights the importance of universal rather than targeted screening. Future directions include measuring the impact of targeted interventions for families who initially screen positive via longitudinal follow-up. Conclusions: Screening for caregiver stress in a primary care medical home for CMC is feasible. As no single variable alone was a predictor of high caregiver stress, universal screening seems to be the most appropriate strategy to capture all families at the highest risk.

## 1. Introduction

Children with medical complexity (CMC), a subset of patients with special healthcare needs, are defined as children with multiple chronic health conditions that affect multiple organ systems and result in extremely high healthcare utilization, as well as a reliance on medical technology [1,2]. These children comprise about one percent of the pediatric population but account for about one third of all pediatric healthcare spending, and this number is projected to increase over time [1,3,4]. The Complex Care Program (CCP) at the Children’s National Hospital provides primary care and care coordination for approximately 800 CMC and their siblings, ages 0–22, within a large academic primary care Medical Home-certified practice in Washington, DC. Though there are many models and definitions of medical complexity, including the diagnosis-related group (DRG) weight, which is a validated metric that quantifies clinical acuity and resource utilization, for our study, we included all children who met the criteria to be followed in the CCP at CNMC [5]. Youth are enrolled in our program if they are followed by three or more medical subspecialties, have frequent hospitalizations, and are dependent on technology such as a G-tube, tracheostomy, or ventilator to sustain life. A primary behavioral health diagnosis (e.g., autism) does not confer eligibility. Approximately 70% of patients are publicly insured.

The caregivers of CMC have unique needs and stressors compared to the guardians of children without complex needs. A caregiver is defined as any individual who coordinates and participates in unpaid care and excludes nurses and professional paid caregivers. The financial stress of caring for CMC is well documented in the literature; studies using national datasets have shown that over half of families with CMC report significant financial stressors and high rates of workforce attrition because of their child’s health, exacerbating financial stress felt by these families [2,6,7]. The extent of unmet needs across income level and insurance status for CMC has led some researchers to suggest that the medical complexity itself may be an independent risk factor for health inequity [8,9]. Studies looking specifically at caregiver stress scales have been replicated in various patient populations globally, all noting increased stress scores among the parents of CMC [10,11,12]. One notable finding of a recent study is that increased psychosocial stress risk scores were not significantly correlated with family demographics [13].

While many studies have noted these unique stressors, and interventions to meet these needs have been undertaken in novel ways, the role of universal screening for caregiver stress using a previously validated tool within the outpatient primary care medical home for children with medical complexity has not been explored in the literature [14]. The medical home is an ideal setting for screening given the longitudinal relationship between the provider and family and the ability to advocate for additional resources and support in the home as needed. As part of routine care, we administered the validated UW Caregiver Stress Scale (UW-CSS) at clinic visits from 7 January 2022 through 30 June 2023 [15].

## 2. Materials and Methods

### 2.1. Study Design

This single-center retrospective study analyzed the results of the pilot implementation of caregiver stress screening surveys collected from a sample of 125 caregivers of children (ages 0–22) who received primary care in the CCP at the Children’s National Hospital. The goal of the analysis was both to evaluate the efficacy and feasibility of screening for caregiver stress during routine appointments, as well as to determine trends, if any existed, between caregiver stress and demographic variables, particularly socioeconomic status.

### 2.2. Study Population

English and Spanish-speaking families who are enrolled in the CCP at the Children’s National Hospital and presented for well-child or care coordination visits during the study period were eligible for completion of the survey.

### 2.3. Materials

The study examined the implementation of caregiver stress screening as a standard of care for all patients in the CCP presenting for routine health maintenance visits. All families presenting for a routine health maintenance visit were given paper copies of the UW-CSS by clinical operations representatives during appointment check-in [13]. All caregivers who obtained elevated scores were referred to the CCP-designated social worker, psychologist, or nursing case manager. The form was scored and documented in the patient’s clinical encounter by their medical provider so that follow-up could be provided by future providers on an individual basis.

The University of Washington Caregiver Stress Scale (UW-CSS) 8-item Short Form V. 2.0 is a tool designed to assess stress levels in caregivers of children under 18 years old. The UW-CSS was developed in a sample of adults (18 years or older) living in the United States who were caring for a child under 18 years of age with either epileptic encephalopathies, Down Syndrome, muscular dystrophy, or no specific healthcare needs to compare scores. The UW-CSS can reliably measure caregiver stress across different populations and settings, including epilepsy, seizure, and general community population patients [14]. The UW-CSS assesses the subdomains of caregiver stress, including time to take care of one’s own health, fatigue, sleep quality, emotional health, relationship quality with others, and family finances. Raw scores are transposed into T-scores with a mean of 50 and a standard deviation of 10. T-scores above 55 are considered clinically elevated. Caregiver stress is a current standard of care that was decided by our team previously to identify parents in need of additional social or psychological support more equitably.

All demographic data were retrospectively collected from the patient’s chart. The demographic data extracted included age, race, ethnicity, insurance provider, address, zip code, primary underlying medical status, number of medications, and technology support status. Study data were entered using REDCap electronic data capture tools hosted at the Children’s National Hospital. Research Electronic Data Capture (REDCap) is a secure, web-based application designed to support data capture for research studies, providing (1) an intuitive interface for validated data entry; (2) audit trails for tracking data manipulation and export procedures; (3) automated export procedures for seamless data downloads to common statistical packages; and (4) procedures for importing data from external sources [16].

Using the demographics extracted from the chart, we identified and linked patient records to their corresponding childhood opportunity index (COI) based on residential addresses. The COI was used as a proxy for the patient’s socioeconomic position [17]. The COI is a standardized composite measure of neighborhood opportunity across three domains: education, health and environment, and social and economic resources, which are predictive of child and adult health outcomes as well as socioeconomic mobility. The COI 3.0 aggregates 44 indicators at the census-tract level, with higher scores indicating greater opportunity. The COI has been shown to outperform several other composite indices, looking at outcomes of health and longevity both in children and adults, which is documented in the COI technical report [18]. The COI is arranged into quintiles of opportunity (very low, low, moderate, high, and very high). A higher childhood opportunity index (COI) score indicates a greater level of neighborhood opportunity for children, and it reflects better access to resources, services, and environmental conditions that support child development, health, and well-being.

### 2.4. Outcome Measures

The primary aim of the study was to identify whether socioeconomic status, as approximated by the childhood opportunity index, correlated positively or negatively with caregiver stress scores. For secondary outcomes, we examined whether other variables (age, race, ethnicity, language, insurance provider) were associated with caregiver stress levels. Furthermore, a secondary chart review was completed for the highest decile of CSS scores and lowest decile of CSS scores from the dataset to determine if there were additional factors within the patient medical record that predicted caregiver stress. These variables included technology support status, annual number of hospitalizations, and yearly subspecialty appointments.

### 2.5. Statistical Analysis

A simple linear regression analysis of the caregiver stress score variable was performed using the childhood opportunity index (COI) as well as key demographic variables. Visual associations were inspected with side-by-side box plots for COI as well as a scatterplot for age. For secondary analysis, additional variables of the highest and lowest deciles of caregiver stress scores were statistically compared with the chi-square test, Fisher’s Exact Test for categorical variables, or with the Mann–Whitney U Test for quantitative/ordinal data. All analyses were performed in SAS V9.4 (Cary, NC, USA), and graphs were produced in R 4.41. Statistical tests were two-sided with a priori alpha of 0.05.

### 2.6. Ethical Considerations

A waiver of informed consent was requested and granted during the Institutional Review Board (IRB) approval process. Our research involved no more than minimal risk to the subject, and UW-CSS collection was already part of our routine clinical care and does not provide a new intervention or practice. The use of protected health information (PHI) does not present more than minimal risk to the privacy of the individual subjects as it does not extend beyond what is currently carried out in our clinic in administering screening forms. All PHI data retrospectively queried from medical charts were captured in RedCap, which is a Health Information Portability and Accountability (HIPAA)-compliant database. Without a waiver of consent, the research could not be carried out since the caregiver stress screening is obtained from every patient, and most surveys were completed already as part of routine care. More psychological harm and inequitable referral to intervention may occur if every patient does not complete the protocols as part of routine clinical care.

One potential source of risk in this study is the risk of gathering sensitive social, behavioral, and medical information. If a participant was observed to experience emotional distress during the screening process, the individual was referred to the clinic psychologist to assess for safety. Of note, this scenario has not occurred to date. In addition to minimal risk, families who screened positive received the benefit from the clinical application of these measures and individualized feedback with a trained psychologist or social work provider.

Subject confidentiality was held strictly by the research team. The subject medical record review was limited to the elements needed to complete the study. Only authorized HIPAA and Collaborative Institutional Training Initiative (CITI)-trained study team members were allowed to extract research data from medical records and enter it into REDCap^®^. No direct subject identifiers were entered into REDCap^®^. Each subject was assigned a unique study number. A master list linking the unique study number to the human subject MRN was maintained in a separate, secure Excel document.

## 3. Results

### 3.1. Overview

**Figure 1:**
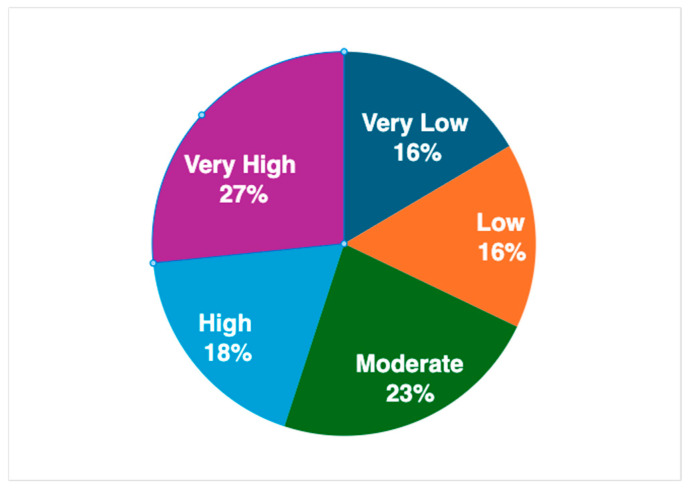
The childhood opportunity index stratifications in the study population.

In total, of the 125 surveys that were collected for analysis in this pilot study, 109 surveys were unique after accounting for duplicate or sibling encounters. In these cases, the caregiver stress scores were averaged between the two encounters. The demographics of our study population are shown in Table 1. The mean caregiver stress T-score in our population was within the average range (M = 48.8, SD = 13.9), with a range from 27 to 77 (Table 2). The sample in this study evenly represented neighborhoods (Figure 1) from very low to very high childhood opportunity indices: very low (*n* = 18, 16.5%), low (*n* = 17, 15.6%), moderate (*n* = 25, 22.9%), high (*n* = 20, 18.3%), and very high (*n* = 29, 26.6%).

### 3.2. No Correlation Between Caregiver Stress and COI

**Figure 2:**
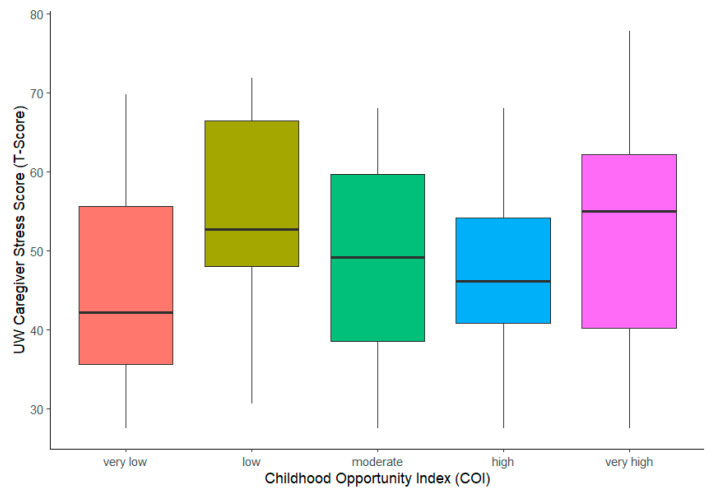
Primary outcome: caregiver stress score vs. childhood opportunity index.

Our data found no correlation between caregiver stress scores and the childhood opportunity index (Figure 2). The mean CSS scores obtained using the COI were the following: very low = 41.4 (0–64.8), low = 53 (30.7–71.9), moderate = 49.8 (27.5–68), high = 46.2 (27.5–68), and very high = 52.2 (27.5–77.8). The overall F-Test *p*-value using the COI was 0.25 (Table 3a), indicating that the COI was not significantly associated with CSS in the sample. Additionally, parameter estimates—by level—are presented with their corresponding *p*-value (Table 3b). Using the “very low” COI as the reference, none of the higher COIs had statistically different levels of caregiver stress.

### 3.3. Correlation Between CSS and Other Demographics (Age, Race, Ethnicity, Language, Insurance)

**Figure 3:**
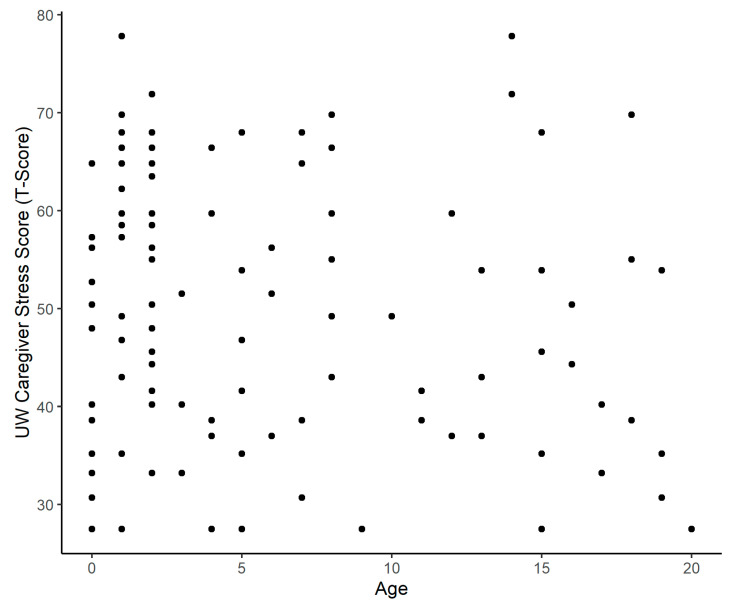
Caregiver stress by age of child.

The mean age of the children whose caregivers were surveyed in this study was 5.5 years, with a median of 2 years. Though it seemed that mean caregiver stress may inversely correlate with age, our results were not statistically significant (*p* = 0.201) (Figure 3). Additionally, there was no statistical significance between race, ethnicity, languages spoken, or insurance status (Table 3a,b).

### 3.4. Caregiver Stress Did Not Correlate with Other Patient Variables

**Figure 4:**
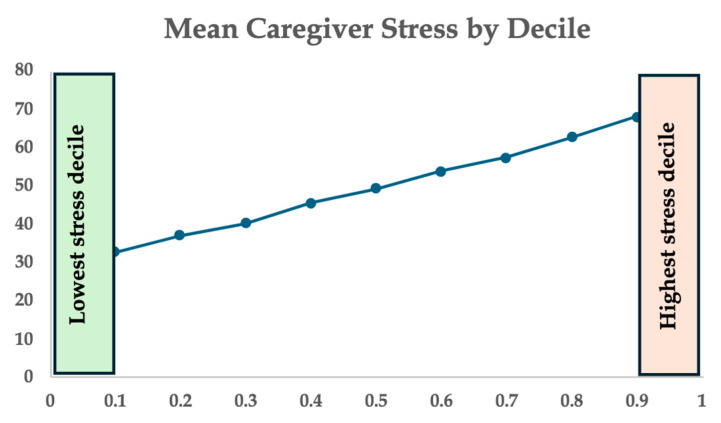
Mean caregiver stress scores by decile. The highest and lowest groups that underwent secondary analysis are highlighted.

Secondary analysis was performed in the top and bottom decile of patients sorted by caregiver stress score (Figure 4). A summary of the comparative findings between the highest and lowest caregiver stress groups is shown in Table 4. We examined the number of hospitalizations and the number of subspeciality appointments per year, as well as the number of daily medications and technology support to compare between the highest and lowest stress groups. Both groups had similar rates of hospitalizations, with a median of 0.5 hospitalizations per year in the lowest caregiver stress records compared with a median of 0 hospitalizations per year for the highest caregiver stress scores (*p*-value = 0.942). The number of subspecialty appointments in 12 months was also not statistically different between the two groups, with medians of 10.5 visits per year in the lowest stress group compared to 8.5 visits in the highest caregiver stress group (*p*-value = 0.466). There was overall higher technology support in the highest caregiver stress group compared to lowest caregiver stress: 92% vs. 60% feeding tube support (*p*-value = 0.135), 45 vs. 20% tracheostomy (*p*-value 0.381), 18 vs. 0% ventilator support (*p*-value = 0.481). Additionally, the higher caregiver stress group also took care of children on a higher number of daily meds (42% on 6+ meds in the high stress group, compared with 90% on <5 meds in the lowest caregiver stress group (*p*-value = 0.162)).

## 4. Discussion

Screening for caregiver stress in our medical home yielded several unexpected results. In our population of children with medical complexity, we found a mean caregiver stress score that was similar to the community population sample that the UW-CSS was based on in validity testing, which included caregivers with and without medically complex children [19]. This is surprising, as we might have expected higher caregiver stress in medically complex children as frequently described previously [12,14,20]. Social desirability bias may have played a role in this finding.

Furthermore, we found no difference in the levels of stress among caregivers based on economic status as measured using the COI. In fact, in comparing the COI of the highest and lowest stress scores, we found that the average COI was higher (indicating higher economic advantage) in the highest stress score group, though the result was not statistically significant (Table 4). Economic status and opportunity are risk factors that have been associated with many mental health outcomes, including anxiety and depression, which are interrelated to caregiver stress. However, there is a growing body of literature suggesting that this relationship may be more complicated. Similar findings were noted in a recent study of CMC caregivers, where an inverse relationship was found between self-reported mental health and socioeconomic status [13]. Similarly, Prieto et al. showed a significant positive correlation between family income and caregiver burden as well as a positive association between the educational level of caregivers and caregiver burden [21]. These results highlight the importance of universal rather than targeted screening.

Additionally, we looked at other variables associated with higher or lower levels of caregiver stress. Caregivers of younger children were oversampled in our patient population due to the frequency of primary care visits in that age group. We note a trend toward higher stress levels at younger ages, though this trend was not statistically significant in our study. It is not surprising that new parents adapting to care for medically complex children may be at higher risk of stress early on. Specific challenges include navigating the healthcare system for the first time, identifying all providers/subspecialists, and having appropriate plans of care for children who are growing and developing at a high rate between the ages of 0–5, as well as setting new expectations for their life and their future. It is reasonable that, over time, these challenges are mitigated as children enter school and begin to receive services and have additional caregivers/providers throughout the day, which may take the burden off the primary caregiver. The inverse relationship between the child’s age, duration of child’s disease, and duration of caregiving to caregiver burden has been previously demonstrated and attributed to the ability of caregivers to develop coping mechanisms and caregiving-induced limitations over time [22]. We did find that in our highest stress group, the children showed metrics of higher complexity, as evidenced by more daily medications and higher levels of technological support. Other metrics did not bear out as differences between the two groups. We hypothesized that the number of hospitalizations per year or number of subspecialty appointments would be associated with higher caregiver stress, as attending appointments or needing frequent appointments would seem to be a marker of illness burden and strain on the family. We did not see any difference between the number of hospitalizations or appointments between the highest and lowest stress groups, which seems to suggest that stress is independent of the need or ability to attend medical appointments or be present for hospitalizations. One qualitative study suggested that caregivers of CMC may uniquely view hospitalizations as a form of personal respite, which could contribute to our findings [2].

### 4.1. Limitations

Our study had several limitations. One major limitation of the study is that the only survey collected was the CSS; all other data were collected in chart review. As such, we used COI as the sole proxy for economic opportunity and financial burden based on demographics found in the chart review and did not ask specific Social Drivers of Health (SDOH) questions. Secondly, our sample size was small and taken from one pediatric institution within a large metropolitan complex, which limits its generalizability to other locales and hospital systems around the U.S. in which access to pediatric medical care and support may differ. Thirdly, we did not collect caregiver stress screeners from a comparative sample of parents without CMC to see how the screener performed in our population overall. We only had the capacity to implement caregiver stress screening in our clinic that provides care for children with medical complexity and did not have the infrastructure or funding to carry out large-scale implementation, including psychology or social work follow-up for positive screens in a comparison group.

Additionally, to understand whether there were additional variables that were predictive of higher caregiver stress, we also attempted to compare the following variables through chart review: access to therapeutic services (physical/occupational/speech therapy), the location of therapeutic services (home and school), as well as school accommodations (IEP/504B plan) and additional support (1:1 nurse at school, home nursing support). During the chart review process, it proved difficult to consistently find the up-to-date and relevant information in the medical record to report this data reliably. As a result, we were unable to assess how caregiver stress might be related to these variables, which are undoubtedly important.

### 4.2. Future Directions

Future directions include expanding our study to include a comparative sample of the caregivers of children without medical complexity. We would also like to collect further surveys to continue to explore the relationship between age, appointment, and hospitalization rates and their effects on caregiver stress. We also plan to track and measure the impact of targeted interventions for families who initially screen positive via longitudinal follow-up in the future.

Additionally, an individual caregiver’s support network is likely an important variable associated with overall caregiver stress. It is reasonable to think that a caregiver who shares the burden of caregiving with other family or community members (spouse, relative, nurse/aid) may have a lower stress level. Future directions should also include the implementation of screening tools to assess parent self-efficacy, resilience, and other protective factors against high rates of caregiver stress to further advocate for positive strength-based interventions that may improve the lives of our patients and their caregivers. This may include a separate parent-completed questionnaire performed at the same time as the UW-CSS to gather reliable answers to these important questions.

## 5. Conclusions

Screening for caregiver stress in a primary care medical home for children with medical complexity was feasible and yielded some unexpected findings. No single variable alone was a predictor of high caregiver stress. Universal screening rather than a targeted approach based on perceived risk factors seems to be the most appropriate strategy to capture all families at the highest risk. A special focus and close follow-up with families with young, medically complex children is also advisable.

## Figures and Tables

**Table 1:** Demographics of study population.

Sample Characteristics	Surveys (*n* = 109)
Age of Children	Range 0–20, Mean 5.5, Median 2
0 to 2	55
3 to 5	14
6 to 10	15
11 to 15	14
15+	11
Race	
Black or African American	65, (78.3%)
White	16, (19.3%)
Asian	2, (2.4%)
American Indian or Alaska Native	0, (0.0%)
Hawaiian, Pacific Islander	0, (0.0%)
Ethnicity	
Hispanic/Latino	19, (20.9%)
Non-Hispanic Latino	72, (79.1%)
Language	
English	95, (87.2%)
Spanish	10, (9.2%)
Other	4, (3.7%)
Childhood Opportunity Index	
Very Low	18 (16.5%)
Low	17, (15.6%)
Moderate	25, (22.9%)
High	20, (18.3%)
Very High	29, (26.6%)
Insurance *	
Public	99, (93.4%)
Private	19, (17.9%)
Other	4, (3.8%)

* Not mutually exclusive.

**Table 2:** Summary metrics of caregiver stress scores in study population.

	Summary Metrics		
	CSS Raw	CSS-Scaled Score	T Score
Mean	21.9	49.5	3.4
Median	21	49.2	3.2
Max	40	77.8	4.9
Min	8	27.5	2.9
Percentiles			
0.1	9.8	32.7	2.9
0.25	13	38.6	3
0.5	21	49.2	3.2
0.75	30	59.7	3.5
0.9	36	68	4.2
0.95	37	69.8	4.9

**Table 3:** (**a**) Linear regression of caregiver stress vs. other demographics. (**b**) Statistical analysis of caregiver stress vs. other demographics using reference values.

(**a**)			
	R2	F-Value	*p*-value
COI	0.050	1.37	0.248
Age	0.015	1.66	0.201
Race	0.006	0.25	0.776
Ethnicity	0.006	0.56	0.457
Language	0.006	0.31	0.732
Insurance	0.009	0.46	0.630
(**b**)			
	Beta (SE)		*p*-value
COI			
Very Low	REF		Ref
Low	7.66 (4.25)		0.087
Moderate	4.42 (4.05)		0.277
High	0.85 (4.25)		0.842
Very High	6.80 (3.93)		0.087
Age	−0.273		0.201
Race			
White	REF		REF
Black	2.04 (3.54)		0.566
Asian	−2.18 (9.53)		0.820
Ethnicity			
Hispanic	−2.56 (3.42)		0.457
Language			
English	REF		REF
Spanish	−0.79 (4.41)		0.858
Other	5.14 (6.77)		0.450
Insurance			
Private	REF		REF
Public	0.96 (3.85)		0.803
Other	−5.31 (7.42)		0.476

**Table 4:** Trends in top and bottom stress deciles of caregiver stress.

	Lowest Stress Decile	Highest Stress Decile	*p*-Values
COI, Median (IQR)	3 (1–4)	4 (2–5)	0.190
Hospitalizations, Median (IQR)	0.5 (0–1)	0 (0–1.5)	0.942
Subspecialty Appointments, Median (IQR)	10.5 (7–11)	8.5 (6–10.5)	0.466
Tech Support			
Feeding Tube (NG/GT)	60%	92%	0.135
Tracheostomy	20%	42%	0.381
Ventilator	0	17%	0.481
Daily Meds			
0–5	90%	58%	0.162
>=6	10%	42%	

## Data Availability

The raw data supporting the conclusions of this article is not publicly available due to privacy considerations but will be made available by the authors upon request.

## Abbreviations

The following abbreviations are used in this manuscript:

CMC	Children with Medical Complexity
CCP	Complex Care Program
UW-CSS	University of Washington Caregiver Stress Scale 8-item Short Form V. 2.0.
COI	Childhood Opportunity Index
PHI	Protected Health Information
HIPAA	Health Information Portability and Accountability Act
CITI	Collaborative Institutional Training Initiative
SDOH	Social Drivers of Health
